# A Novel Binding Mode Reveals Two Distinct Classes of NMDA Receptor GluN2B-selective Antagonists[Fn FN5]

**DOI:** 10.1124/mol.115.103036

**Published:** 2016-05

**Authors:** David Stroebel, Derek L. Buhl, John D. Knafels, Pranab K. Chanda, Michael Green, Simone Sciabola, Laetitia Mony, Pierre Paoletti, Jayvardhan Pandit

**Affiliations:** Ecole Normale Supérieure, PSL Research University, CNRS, INSERM, Institut de Biologie de l'École Normale Supérieure (IBENS), Paris, France (D.S., L.M., P.P.); Pfizer Worldwide Research and Development, Cambridge, Massachusetts (D.L.B., M.G., S.S.); and Pfizer Worldwide Research and Development, Groton, Connecticut (J.D.K., P.K.C., J.P.)

## Abstract

*N*-methyl-d-aspartate receptors (NMDARs) are glutamate-gated ion channels that play key roles in brain physiology and pathology. Because numerous pathologic conditions involve NMDAR overactivation, subunit-selective antagonists hold strong therapeutic potential, although clinical successes remain limited. Among the most promising NMDAR-targeting drugs are allosteric inhibitors of GluN2B-containing receptors. Since the discovery of ifenprodil, a range of GluN2B-selective compounds with strikingly different structural motifs have been identified. This molecular diversity raises the possibility of distinct binding sites, although supporting data are lacking. Using X-ray crystallography, we show that EVT-101, a GluN2B antagonist structurally unrelated to the classic phenylethanolamine pharmacophore, binds at the same GluN1/GluN2B dimer interface as ifenprodil but adopts a remarkably different binding mode involving a distinct subcavity and receptor interactions. Mutagenesis experiments demonstrate that this novel binding site is physiologically relevant. Moreover, in silico docking unveils that GluN2B-selective antagonists broadly divide into two distinct classes according to binding pose. These data widen the allosteric and pharmacological landscape of NMDARs and offer a renewed structural framework for designing next-generation GluN2B antagonists with therapeutic value for brain disorders.

## Graphical abstract

**Figure:**
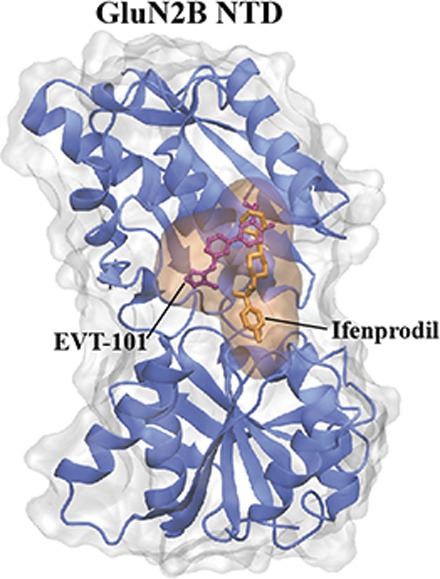

## Introduction

*N*-methyl-d-aspartate receptors (NMDARs) are ionotropic glutamate receptors widely expressed in the central nervous system that mediate excitatory postsynaptic signaling (Traynelis et al., 2010). These receptors are essential for normal physiologic processes such as neuronal development, synaptic plasticity, and learning and memory. NMDARs are also implicated in a plethora of brain disorders, thus receiving intense interest as potential therapeutic targets. Conditions including ischemic damage, chronic pain, depression, and major neurodegenerative disorders, have been suggested to involve overactivation of NMDAR activity (Traynelis et al., 2010; Paoletti et al., 2013). NMDAR antagonists are therefore thought to hold strong therapeutic potential, although successes in the clinic have been limited (Kalia et al., 2008; Mony et al., 2009a; Paoletti et al., 2013).

**Fig. 1.**
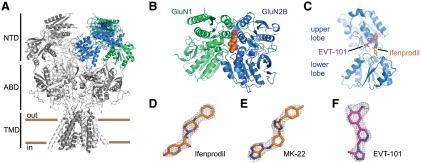
X-ray crystal structure of the GluN1/GluN2B NTD dimer in complex with EVT-101. (A) Structure of the tetrameric GluN1/GluN2B receptors (Karakas and Furukawa, 2014; Lee et al., 2014). GluN1 subunits are in dark gray and GluN2B subunits in pale gray. One NTD heterodimer is highlighted (GluN1 in green, GluN2B in blue). NTD, N-terminal domain; ABD, agonist-binding domain; TMD, transmembrane domain. (B) Structure of the GluN1/GluN2B NTD heterodimer in complex with EVT-101. For comparison purposes, the ifenprodil molecule as seen in the GluN1/GluN2B NTD-ifenprodil complex is superimposed. The two ligands shown in sphere representation (ifenprodil in orange, EVT-101 in purple) sit at the heterodimer interface. (C) Side view (rotated 90°) with the GluN1 NTD removed and the ligands shown in stick representation. (D–F) Difference electron density maps (mFo-DFc) for ifenprodil, MK-22, and EVT-101 contoured at 3.0 *σ*.

NMDARs are heterotetramers usually associating two GluN1 and two GluN2 subunits (Traynelis et al., 2010; Paoletti et al., 2013). The GluN2 subunits, of which there are four subtypes (A–D), control a wide range of the receptor’s functional properties and are differentially expressed throughout the central nervous system (Monyer et al., 1994; Sheng et al., 1994; Paoletti, 2011). At the structural level, NMDARs form massive molecular complexes (>550 kDa) with a typical layered organization shared with other ionotropic glutamate receptors, which consists of a layer of N-terminal domains (NTDs) and a layer of agonist-binding domains directly connected to the transmembrane pore region (Fig. 1A) (Karakas and Furukawa, 2014; Lee et al., 2014). The NTDs endow NMDARs with a unique capacity for allosteric modulation, harboring several binding sites for small molecule ligands that act as subunit-specific allosteric modulators of ion channel activity (Zhu and Paoletti, 2015). In particular, the GluN2B NTD confers a rich pharmacology with distinct recognition sites for both endogenous and exogenous allosteric inhibitors and potentiators (Karakas et al., 2009, 2011; Mony et al., 2009a, 2011). Among these ligands are ifenprodil and derivatives, a large family of synthetic compounds that act as highly selective noncompetitive antagonists of GluN2B-containing NMDARs (hereafter termed GluN2B receptors) (Williams, 1993; Chenard and Menniti, 1999; Nikam and Meltzer, 2002; Borza and Domany, 2006; Layton et al., 2006). Ifenprodil and related compounds are widely used for pharmacological profiling of native NMDARs and have served as lead compounds in therapeutic applications (Mony et al., 2009a). Interestingly, GluN2B-selective antagonists have shown encouraging results in a number of clinical trials with a better side effect profile than pan-NMDAR antagonists such as ketamine (Preskorn et al., 2008; Mony et al., 2009a; Ibrahim et al., 2012). This enhanced tolerability likely stems from a combination of subunit selectivity, such that non-GluN2B receptors are spared, and an atypical mode of action, such that strongly activated receptors are preferentially inhibited (Kew et al., 1996; Mott et al., 1998; Yuan et al., 2015). Moreover, in vitro and in vivo evidence suggests that GluN2B receptors preferentially couple to prodeath signaling pathways (Martel et al., 2012), although this remains debated (Paoletti et al., 2013).

Since the discovery that ifenprodil selectively inhibits GluN2B receptors (Williams, 1993), a vast range of GluN2B-selective antagonists have been developed. Many, such as CP-101,606 (Traxoprodil; Chenard et al., 1995), Ro25-6981 (Fischer et al., 1997), or besonprodil (Chizh et al., 2001), share the same phenylethanolamine scaffold as ifenprodil. Others, including a number of highly potent and orally active GluN2B antagonists such as EVT-101 (Kemp and Tasker, 2009) and to a lesser extent MK-22 (Layton et al., 2011), bear strikingly different structural motifs, thus questioning whether these compounds adopt a similar binding mode to ifenprodil. Crystal structures recently established the existence of a "phenylethanolamine binding site" at a dimer interface between GluN1 and GluN2B NTDs (Karakas et al., 2011; Karakas and Furukawa, 2014; Lee et al., 2014). To better understand the protein-ligand interactions necessary for potency and selectivity of other nonphenylethanolamine scaffolds, we used a back-soaking protocol to solve the structures of EVT-101 and MK-22 in complex with the GluN1/GluN2B NTD heterodimer at a 2.8- to 3.0-Å resolution. We report here that although the binding of MK-22 is essentially superimposable on that of ifenprodil, EVT-101 occupies a distinct cavity with only partial overlap and makes new interactions with the pyridazine and imidazole groups. By performing mutagenesis experiments on full-length receptors expressed in *Xenopus* oocytes, we also provide evidence that this novel binding pocket is functionally relevant. Finally, by computing protein-ligand fingerprints based on an array of structurally diverse GluN2B-selective antagonists, we show that these compounds cluster in (at least) two different classes according to their binding pose at the GluN1/GluN2B dimer interface. Our combined structural, functional, and modeling data reveal that the GluN1/GluN2B NTD dimer cavity is wide enough to accommodate a range of structurally diverse ligands that adopt distinct binding modes. In addition to providing a new look on GluN2B pharmacology, this work may provide insight on how to screen novel therapeutically relevant compounds and differentially modulate NMDAR populations.

## Materials and Methods

### 

#### Compounds.

EVT-101 [5-(3-(difluoromethyl)-4-fluorophenyl)-3-((2-methyl-1H-imidazol-1-yl)methyl)pyridazine] and MK-22 [*N*-[(1S,3S)-3-[3-(4-methylbenzyl)-1,2,4-oxadiazol-5-yl]cyclopentyl]-1H-pyrazolo[3,4-day]pyrimidin-4-amine] were synthesized in-house at Pfizer (Groton, CT) following the procedure described in Kemp and Tasker (2009) and Layton et al. (2011), respectively. EVT-101 is achiral (i.e., possessing no stereocenters), whereas MK-22 is a pure enantiomer, being synthesized from chiral starting material. Ifenprodil used at Pfizer for crystallographic studies was synthesized according to Chenard et al. (1991) and references therein. Ifenprodil used at Institut de Biologie de l'École Normale Supérieure (Paris, France) for electrophysiological recordings was obtained from Synthélabo (Bagneux, France) (a generous gift from B. Scatton). The synthesis of ifenprodil, which contains two stereocenters, is diastereoselective, and the final product is a racemic mixture composed of the (+) and (−) enantiomers of the erythro diasteroisomer. In our crystals, all ligand densities were clearly defined and could be fitted without ambiguity. For ifenprodil, the observed density was best assigned to a single enantiomer [the (+) enantiomer, which is slightly more potent than the (−) form (Avenet et al., 1996)].

#### Protein Expression and Purification.

Expression, purification and crystallization protocols are based on those described previously (Karakas et al., 2011). A dual expression construct was made containing the *Xenopus laevis* GluN1 NTD (Met 1 to Glu 408) containing Cys22Ser, Asn61Gln, and Asn371Gln mutations, a C-terminal thrombin cleavage site, followed by a FLAG tag and the human GluN2B NTD (Ser 31 to Met 394) containing an Asn348Asp mutation with a N-terminal human placental alkaline phosphatase signal sequence, followed by a FLAG tag and a thrombin cleavage site. These were coexpressed using the pFastBac Dual vector in Sf9 cells and secreted into the media. A total of 10 liters of media was neutralized and batch bound with 10 ml of FLAG resin overnight at 4°C. The resin was collected, washed with 20 mM Tris-HCl (pH 8.0) and 200 mM NaCl, and eluted with FLAG peptide. The FLAG pool was incubated with 10 *µ*M ifenprodil overnight. Ifenprodil (1 *µ*M) was kept in all subsequent buffers. After concentration, size exclusion chromatography was used to separate the GluN1-GluN2B heterodimer from excess GluN1. The heterodimer was deglycosylated overnight with endoglycosidase F1, followed by tag cleavage with thrombin and another round of size exclusion chromatography in 20 mM Tris-HCl (pH 8.0), 200 mM NaCl, and 1 *µ*M ifenprodil. The GluN1-GluN2B NTD complex with ifenprodil was concentrated to 8 mg/ml. Sitting-drop vapor diffusion crystallization experiments were set up by mixing a 2:1 ratio of protein with a reservoir solution containing 0.1 M HEPES pH 6.8–7.2 and 3.5–3.7 M sodium formate. Crystals appeared in 2 days and were cryoprotected by quickly dipping into 5.0 M sodium formate and 0.1 M HEPES (pH 6.8). To obtain complexes with other ligands, crystals were soaked in solutions containing 2.5 mM of EVT-101 (Kemp and Tasker, 2009) or MK-22 (Layton et al., 2011) in cryoprotectant solution for 1–3 days.

#### X-ray Data Collection and Structure Solution.

X-ray diffraction data were collected at 100 K with radiation of wavelength 1.0 Å at the Advanced Photon Source at Argonne National Laboratory (Lemont, IL), beamline 17ID. The diffraction data were processed with autoPROC (Global Phasing Limited, Cambridge, UK) (Vonrhein et al., 2011). Further data manipulations were carried out using the CCP4 program suite [Collaborative Computational Project (CCP4, Didcot, UK), Number 4, 1994]. Initial phases for the ifenprodil structure were generated by rigid-body refinement with the coordinates from Protein Data Bank entry 3QEL (Karakas et al., 2011) after removing all water molecules and ligand atoms. After fitting the ligand into unambiguous difference density, all-atom refinement was carried using the program autoBUSTER (Global Phasing Ltd. Cambridge, UK). This refined protein model was used as the starting model to generate phases for the MK-22 and EVT-101 soaks. The data and refinement statistics are summarized in Supplemental Table 1. Coordinates and structure factors have been deposited in the Protein Data Bank with accession codes 5EWJ, 5EWM, and 5EWL for the ifenprodil, EVT-101, and MK-22 complexes, respectively.

#### Cavity Size Measurements.

Volumes of pockets (or cavities) were estimated using POVME (Durrant et al., 2011). We first modeled the entire cavity corresponding to the empty space at the dimerization interface between the two NTDs when inhibitor molecules are removed. We based the cavity sampling limits on two "inclusion spheres" (12 Å: 83 4 -32; 5 Å: 91 12 -31) chosen to include the entire binding pocket and 18 "exclusion spheres" designed to remove cavities distinct from the inhibitor binding pocket or volumes corresponding to the exterior of the protein structure. Gridspacing was 1.0 and padding 1.09. The volume that ifenprodil and EVT-101 occupy in the previously defined pocket was then calculated using the function "neighbors of selected residues" in SPDB Viewer (Guex and Peitsch, 1997), with a 1.6 Å search radius around each compound. The "common pocket" volume was estimated by calculating the arithmetic mean between the volume occupied by ifenprodil in the EVT-101 cavity and vice versa.

#### In Silico Docking Experiments.

The 3D coordinates of the GluN2B receptor subunit were taken from the in-house crystal structure in complex with ifenprodil. The protein preparation workflow within Maestro [Schrödinger Release 2015-3: Maestro, version 10.3, Schrödinger, LLC, New York, NY, 2015 (Sastry et al., 2013)] was used to prepare the protein in a form that is suitable for molecular modeling calculations. The ligands were titrated at neutral pH using MoKa (Milletti et al., 2007) and then converted to 3D using Corina (Sadowski et al., 1994). The Glide standard precision (small-molecule drug discovery suite 2015-3, 2015; Friesner et al., 2004) protocol was used to dock the ligands into the protein active site. The protein was kept rigid and the ligands flexible. The standard protocol was adjusted to improve ligand conformational sampling and initial poses generation. The top five docking poses for each ligand were kept for further processing. The best pose for each ligand based on energy and visual inspection was then locally refined using the Glide extra precision protocol (Friesner et al., 2006). In addition, a matrix was generated containing the minimum distances between each ligand and the following amino acids (A75, A106, A108, A109, A110, A112, A113, A115, A131, A132, A133, A134, A135, B78, B82, B106, B107, B110, B111, B113, B114, B115, B134, B135, B136, B137, B174, B175, B176, B177, B207, B233, B235, B236) and water molecules (W100, W134, W304). This information was used to summarize the differences in binding modes among the docked ligands. The rows (ligands) and columns (amino acids interaction distance) of this matrix were represented as a clustered heatmap using euclidean distance as metric in combination with agglomerative hierarchical clustering. This analysis was performed within the RStudio environment (Friesner et al., 2006) using the Pheatmap package (https://cran.r-project.org/web/packages/pheatmap/index.html).

#### Electrophysiology.

Electrophysiology experiments were performed using rodent NMDARs. The pcDNA3-based expression plasmids for the rat GluN1-1a subunit (named GluN1 herein) and the mouse GluN2B subunit have been described previously (Mony et al., 2011). Site-directed mutagenesis was performed using QuikChange (Stratagene, La Jolla, CA), and the presence of the mutation was verified by DNA sequencing.

Recombinant NMDARs were expressed in *Xenopus laevis* oocytes after nuclear coinjection of GluN1 and GluN2 cDNAs (at 10 ng/*µ*l each, ratio 1:1). Oocytes were prepared, injected, voltage-clamped, and superfused as described previously (Mony et al., 2011). The superfusing external solution contained (in mM): 100 NaCl, 0.3 BaCl_2_, 5 HEPES, and 0.01 diethylenetriamine-pentaacetic acid (pH adjusted to 7.3 with KOH). Diethylenetriamine-pentaacetic acid (10 *µ*M) was added to chelate trace amounts of heavy metals [including zinc (Paoletti et al., 1997)]. NMDAR-mediated currents were induced by applying saturating concentrations of glutamate and glycine (Glu + Gly, 100 *µ*M each). Currents were recorded at a holding potential of −60 mV, and experiments were done at room temperature. Error bars represent standard deviation unless otherwise stated. Compound (ifenprodil, EVT-101) dose-response curves were fitted with the following Hill equation: I_rel_ = 1-a/([1+(IC_50_/[C])^nH^], where I_rel_ is the mean current normalized to the current obtained in the absence of compound, [C] is the compound concentration, n_H_ is the Hill coefficient and a the maximal inhibition. For certain fits, the maximal inhibition "a" was fixed to 1.0 (see Table 1). MK-801 inhibition kinetics experiments and analysis were performed as previously described (Zhu et al., 2013). MK-801 was used at a concentration of 30 nM.

**TABLE 1:** Effects of GluN1 and GluN2B NTD mutations on EVT-101 and ifenprodil sensitivity Both IC_50_ values and levels of maximal inhibition (Max inhib) deduced from the curve fits are given (see *Materials and Methods*).

GluN1	GluN2B	Relative MK-801		Ifenprodil				EVT-101			
		*τ*_on_	*n*	IC_50_	IC_50_ ratio (mutant/wt)	Max inhib	*n*	IC_50_	IC_50_ ratio (mutant/wt)	Max inhib	*n*
				*nM*				*nM*			
wt	wt	1.0	(9)	130 ± 4	1.0	0.95 ± 0.01	(12)	12 ± 0.2	1.0	0.9 ± 0.01	(12)
Y109C	wt	1.1	(3)	856 ± 122	6.6	1.0*	(4)	7300 ± 260	608	1.0*	(3)
Y109S	wt	0.7	(3)	224 ± 36	1.7	0.97 ± 0.07	(4)	9618 ± 700	800	1.0*	(3)
I133A	wt	2.9	(5)	80 ± 7	0.6	0.69 ± 0.02	(3)	52 ± 4	4.3	0.65 ± 0.01	(4)
I133C	wt	0.6	(4)	1400 ± 104	11	1.0*	(4)	62 ± 22	5.2	0.48 ± 0.03	(3)
I133W	wt	2.6	(6)	32 ± 5	0.3	0.47 ± 0.02	(5)	32 ± 9	2.7	0.54 ± 0.03	(3)
L135A	wt	5.4	(7)	101 ± 9	0.8	0.90 ± 0.03	(5)	25 ± 2	2.0	0.60 ± 0.01	(8)
L135H	wt	6.0	(4)	>10000	>100	1.0*	(3)	71 ± 7	5.9	0.48 ± 0.01	(3)
L135M	wt	2.4	(3)	73 ± 2	0.6	0.86 ± 0.01	(3)	n.d		n.d.	
L135R	wt	3.5	(3)	300 ± 36	2.3	1.0*	(3)	53 ± 6	4.4	0.69 ± 0.02	(3)
L135W	wt	2.1	(9)	767 ± 78	5.9	1.0*	(5)	9 ± 1	0.7	0.86 ± 0.02	(6)
wt	Q110G	0.4	(3)	124 ± 3	1.0	0.99 ± 0.01	(3)	502 ± 14	42	0.97 ± 0.01	(3)
wt	F114S	0.4	(3)	>10000	>100	1.0*	(4)	676 ± 10	56	0.91 ± 0.01	(3)
wt	A135G	3.0	(3)	50 ± 3	0.4	0.80 ± 0.01	(4)	72 ± 7	6.0	0.80 ± 0.02	(3)
wt	A135P	2.1	(4)	587 ± 43	4.5	1.0*	(3)	404 ± 15	34	0.78 ± 0.02	(3)
wt	F176A	1.3	(4)	7500 ± 58	58	1.0*	(4)	11400 ± 1000	950	1.0*	(3)
wt	P177C	2.7	(8)	>10000	>100	1.0*	(6)	75 ± 12	6.2	0.64 ± 0.03	(5)
wt	E236C	0.9	(4)	1800 ± 14	14	1.0*	(4)	332 ± 2	28	0.87 ± 0.01	(4)

wt, wild type; *n*, number of cells; n.d., not determined.

* Indicates maximal inhibition fixed to 1. Errors are expressed as S.D.

## Results and Discussion

To define the location of the MK-22 and EVT-101 binding sites, we conducted crystallographic studies on the GluN1-GluN2B NTD heterodimer. We solved a cocrystal structure of the NTD heterodimer complex with ifenprodil at a 2.8-Å resolution, and then, using a back-soaking protocol (see *Materials and Methods*), determined the structures of the complexes with MK-22 and EVT-101 at a 3.0- and 2.8-Å resolution, respectively (Fig. 1 and Supplemental Fig. 1). All structures were solved by molecular replacement and were refined satisfactorily (Supplemental Table 1). Most notably, in each structure, ligands could be unambiguously modeled into clear difference electron density maps (Fig. 1, D–F), thus allowing clear identification of the compound binding sites.

### 

#### MK-22 and Ifenprodil Bind at the Phenylethanolamine Binding Site.

**Fig. 2.**
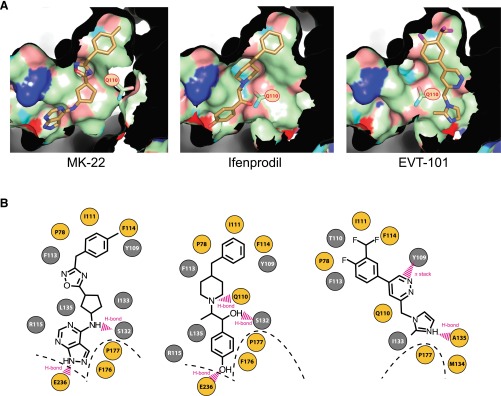
Comparison of the EVT-101, MK-22, and ifenprodil binding sites. (A) Views of the binding pockets of MK-22, ifenprodil, and EVT-101 at the GluN1/GluN2B NTD dimer interface. Lateral views as seen from the GluN2B subunit. Ligands are represented in stick with carbons colored in gold. The color code for the surface of the binding cavities is the following: green for carbons, blue for amines of basic residues, cyan for amines of backbone or polar residues, red for carboxylate groups, and salmon for oxygens of noncarboxylate groups. The amide group shown in stick corresponds to that of residue GluN2B-Q110, which delineates the two subcavities. (B) Contact maps showing residues that interact with MK-22, ifenprodil, and EVT-101. GluN1 and GluN2B residues are shown in gray and yellow, respectively. Amino acids shown in circles are making direct contacts with the ligand. Residues below the dashed line locate in the lower lobe of the GluN2B NTD.

Both the GluN1 and GluN2B NTDs have typical bilobate clamshell-like architectures composed of an upper and lower lobe that adopt a twisted structure, as previously observed (Karakas et al., 2011; Karakas and Furukawa, 2014; Lee et al., 2014). Ifenprodil binds at the heteromeric interface between the two domains and interacts with residues from the upper lobe of the GluN1 NTD and from both the upper and lower lobes of GluN2B NTD (Fig. 1, A–C). Our crystals of the ifenprodil complex are isomorphous with those reported previously (Karakas et al., 2011), and the overall protein structures are identical (root mean square deviation for 700 C*α* atoms of 0.36Å, see Supplemental Table 1). The narrow elongated binding site is occupied by the ligand in an extended conformation (Fig. 2). One end of this site, which nestles the benzylpiperidine moiety of the ifenprodil molecule, is buried in the hydrophobic interface of the upper lobes of the GluN1 and GluN2B NTDs, capped by F114, I82 (GluN2B), and A75, P106 (GluN1). The other end, hosting the phenol moiety, is partially exposed to solvent and makes both polar and hydrophobic interactions with the receptor. Thus, the distal hydroxyl group of the phenol moiety interacts with E236 from the lower lobe of GluN2B, whereas the aromatic ring interacts with a cluster of hydrophobic residues, including GluN1-L135, GluN2B-F176, and GluN2B-P177.

MK-22 occupies the same exact binding site as that of ifenprodil, overlaying perfectly (Supplemental Fig. 1). The terminal benzyl groups on both ligands overlap atom-for-atom, with the extra methyl group on MK-22 extending deeper into the hydrophobic pocket at the upper lobe interface (Fig. 2). At the other end, the nitrogen atoms of the pyrazolo-pyrimidine make the same interactions with water and GluN2B-E236 made by the phenol hydroxyl group of ifenprodil. Similarly, the amine nitrogen in MK-22 makes the same backbone interaction to the carbonyl oxygen of GluN1-S132 as that of the linker hydroxyl in ifenprodil. Interestingly, GluN2B-Q110, which makes a hydrogen bond with the central piperidine nitrogen of ifenprodil, was found to swing away toward the solvent in the MK-22 complex, presumably to avoid an unfavorable interaction between the polar end of the glutamine side chain and the cyclopentyl ring at the center of MK-22 (Fig. 2).

#### EVT-101 Occupies a Different Binding Pocket than Ifenprodil and MK-22.

As for MK-22, the structure of the GluN1-GluN2B NTD dimer solved after soaking with EVT-101 appeared almost perfectly superimposable with that obtained with ifenprodil (root mean square deviation values for any pair of structures ranging from 0.3 to 0.5 Å). However, inspection of the EVT-101 binding site revealed a remarkably different situation with a shared interdomain cavity but little overlap with the ifenprodil and MK-22 binding sites (Fig. 1). Although the hydrophobic ends of all three ligands occupy the same pocket at the NTD upper lobe interface, the remainder of EVT-101 occupies a solvent-exposed groove (or subcavity) departing from the phenylethanolamine binding site (Fig. 2 and Supplemental Fig. 2). This groove is the same that was partially occupied by the side chain of GluN2B-Q110 in the MK-22 complex. Thus, Q110 swings back to the same rotamer as in the ifenprodil structure, although its distal amide moiety does not contact the ligand but rather a water molecule. Conversely, the proximal part of GluN2B-Q110, including the backbone carbonyl and both C*α* and C*β*, lines the binding cavity and provides multiple interactions with EVT-101. Another key interaction for EVT-101 appears to be a *π*-stacking interaction between the central pyridazine ring and GluN1-Y109. The pyridazine moiety of EVT-101 is thus sandwiched between the aromatic ring of GluN1-Y109 and the aliphatic part of the side chain of GluN2B-Q110. The backbone carbonyl of GluN2B-A135 is also ideally placed to directly contact the imidazole ring of the ligand through a hydrogen bond with the protonated nitrogen at position 3 of the imidazole ring [experimental pKa = 6.86, measured using methods described in Shalaeva et al. (2008)]. It is noteworthy that EVT-101, unlike MK-22 and ifenprodil, makes minimal interactions with the lower lobe of the GluN2B NTD (Fig. 2). Van der Waals interactions between the ring of GluN2B-P177 and the distal imidazole moiety (including the methyl substituent) were identified as the only short-distance interactions between EVT-101 and the GluN2B NTD lower lobe. In contrast, ifenprodil and MK-22 contact this part of the receptor through diverse interactions, both polar and hydrophobic, involving multiple residues. Finally, detailed inspection of the crystal structures revealed that protein side chain movements were also visible at the binding sites when comparing EVT-101 and the ifenprodil/MK22 structures, such as the side chains of GluN1-I133 and GluN1-L135, which adopt different rotamers to fill the empty space in the phenylethanolamine binding pocket.

#### Functional Validation of the Novel Binding Pocket Using Structure-Guided Mutagenesis.

Extensive mutagenesis experiments previously identified several residues at the GluN1-GluN2B NTD dimer interface critical for inhibition by ifenprodil and phenylethanolamine derivatives (Gallagher et al., 1996; Masuko et al., 1999; Perin-Dureau et al., 2002; Malherbe et al., 2003; Mony et al., 2009b; Karakas et al., 2011; Burger et al., 2012). To validate the physiologic relevance of the newly identified binding mode of EVT-101, we performed structure-guided mutagenesis and evaluated the sensitivity of the mutant receptors to EVT-101 and ifenprodil using two-electrode voltage-clamp recordings in *Xenopus* oocytes. Given the structural similarities observed for ifenprodil and MK-22, we primarily focused our electrophysiology experiments on ifenprodil and EVT-101. Full dose-response curves were obtained for both compounds, and IC_50_ as well as maximal inhibition values were systematically compared with that of the wild-type receptor (Table 1). For each mutant receptor, we also estimated the maximal level of receptor activity by assessing the inhibition kinetics of MK-801, a selective open-channel blocker classically used to index receptor channel open probability (see Zhu et al., 2013). Based on our crystal structures, we designed a total of 17 mutant receptors encompassing 3 GluN1 positions and 6 GluN2B positions (Table 1). Positions were chosen according to their location and ligand interactions in the respective ifenprodil and EVT-101 binding cavities.

**Fig. 3.**
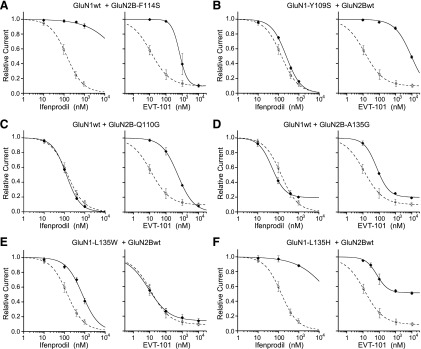
Effects of GluN1 and GluN2B NTD mutations on EVT-101 and ifenprodil sensitivity. Dose-response inhibition curves of GluN1/GluN2B mutant receptors (♦). The dashed curves are the fits of the ifenprodil or EVT-101 dose-response data points obtained on wild-type (wt) receptors (○). The number of cells and the estimated values of IC_50_ and maximal inhibition for each mutant receptor are listed in Table 1. Estimated values of n_H_ are comprised in the range 0.6–1.3 for ifenprodil and 0.7–1.4 for EVT-101. (A) GluN1-F114S/GluN2Bwt. (B) GluN1-Y109C/GluN2Bwt. (C) GluN1wt/GluN2B-Q110G. (D) GluN1wt/GluN2B-A135P. (E) GluN1-L135W/GluN2Bwt. (F) GluN1-L135H/GluN2Bwt. Note that although certain mutations affect the sensitivity to ifenprodil and EVT-101 indiscriminately, others have specific effects for either one of the two ligands. Error bars represent S.D.

We first targeted residue GluN2B-F114, which caps the hydrophobic cavity at the NTD upper-lobe dimer interface where both compounds anchor via their benzyl moiety. As expected, disrupting this hydrophobic lid by introducing a small hydrophilic residue (GluN2B-F114S mutation) resulted in a marked drop of both ifenprodil and EVT-101 sensitivity (>100-fold and 56-fold increase in IC_50_, respectively; Fig. 3A). Similarly, mutating GluN2B-P177, a position of the GluN2B NTD lower lobe that makes atomic contacts with the two ligands, reduced both ifenprodil and EVT-101 potency (GluN2B-P177C mutation; >100-fold and 6-fold increase in IC_50_, respectively). Mutating the nearby position GluN2B-F176 for a small side chain alanine residue also reduced sensitivity to both ifenprodil and EVT-101, the effect on EVT-101 being particularly robust (950-fold increase in IC_50_). This massive effect on EVT-101 sensitivity is somewhat surprising given that GluN2B-F176 is closer to ifenprodil than EVT-101 (nearest distance 3.53 Å versus 5.33 Å, respectively). However, GluN2B-F176 points its side chain straight toward the GluN2B upper lobe NTD *β*4-*β*5 loop, a region critically involved in EVT-101 binding (see below). We suspect the GluN2B-F176A mutation creates a void hydrophobic cavity, thus forcing a change in the local conformation of the GluN2B NTD *β*4-*β*5 loop, which in turn impacts the binding of EVT-101. We believe that similar perturbations of the NTD local structure account for the effect of the GluN2B-E236C mutation, which affected the sensitivity to both compounds.

We next selected several positions based on their close proximity and direct atomic interactions with EVT-101. Disruption of the key *π*-stacking interaction with the central pyridazine ring of EVT-101 by mutating GluN1-Y109 into the nonaromatic and short serine resulted in a drastic decrease in EVT-101 sensitivity (800-fold increase in IC_50_). In contrast, sensitivity to ifenprodil was barely affected (<2-fold change in IC_50_; Fig. 3B, and see also the GluN1-Y109C mutation in Table 1), thus providing strong evidence for the critical importance of residue GluN1-Y109 in mediating EVT-101 inhibitory action. We obtained additional support for the relevance of the EVT-101 binding site by investigating mutations at positions GluN2B-Q110 (*α*2 helix) and GluN2B-A135 (*β*4-*β*5 loop). As described above, both residues line the EVT-101 cavity, making specific contacts with the ligand. Substituting GluN2B-Q110 by a glycine to avoid any side chain-ligand interaction strongly decreased EVT-101 sensitivity (>40-fold increase in IC_50_), whereas ifenprodil sensitivity was totally unaffected (Fig. 3C and Table 1). Similarly, introducing a proline at position GluN2B-A135 to disrupt the backbone interaction with EVT-101 preferentially affected EVT-101 sensitivity (Table 1), whereas introducing a glycine at this position caused a decrease of EVT-101, but not ifenprodil, sensitivity (GluN2B-A135G mutation; Fig. 3D). Thus, in good agreement with the crystal structures, the three mutations GluN1-Y109S, GluN2B-Q110G, and GluN2B-A135G (and to a lesser extent GluN2B-A135P) displayed strong EVT-101-specific phenotypes.

Finally, we targeted two GluN1 residues, I133 and L135, which together with GluN1-Y109 line the GluN1 face of the cavity at the NTD heterodimer interface. Inspection of the crystal structures indicated that GluN1-I133 forms a hydrophobic surface roughly equidistant to both ifenprodil and EVT-101 but not directly contacting the ligands. In contrast, the GluN1-L135 side chain engages multiple atomic contacts with the ifenprodil molecule, but points away from EVT-101. In accordance with these observations, substitutions of GluN1-I133 affected inhibition by both ligands, although the effects were modest and variable according to the nature of the substitution (Table 1). Substitutions at position GluN1-L135 yielded more striking phenotypes. In particular, mutation GluN1-L135W was found to be highly specific for ifenprodil, decreasing sensitivity to this ligand but not to EVT-101 (5.9-fold versus 0.7-fold change in IC_50_, respectively; Fig. 3E). Effects observed with the GluN1-L135H mutation provided further evidence for a critical role of GluN1-L135 in controlling ifenprodil sensitivity. Indeed, although sensitivity to EVT-101 was modestly affected by this mutation (5.9-fold increase in IC_50_), high-affinity ifenprodil inhibition was completely abolished (>100-fold increase in IC_50_) (Fig. 3F). We believe these results indicate that the incorporation of a bulky histidine or tryptophan disrupts ifenprodil binding because of steric clashes. Conversely, the EVT-101 molecule, which resides in a more distant binding pocket, appears to accommodate these receptor modifications more easily. GluN1-L135 mutations also produced marked alterations in the extent of receptor inhibition ("maximal inhibition"; Table 1 and Fig. 3, E and F). Although the mechanistic and structural basis of these plateau effects remain unclear, we speculate that they may be related to the strong "gating effects" produced by these mutations on basal receptor activity (as evidenced by the altered MK-801 inhibition kinetics; Table 1). We note that similar "plateau" phenotypes were previously observed with several others mutations at the NTD dimer interface (Masuko et al., 1999; Karakas et al., 2011), consistent with an important role of this region in mediating allosteric control of the receptor’s downstream gating machinery (Karakas et al., 2011; Zhu et al., 2013, 2014).

#### GluN2B Antagonists Subdivide into Multiple Families According to Their Binding Modes.

Our X-ray crystallography and mutagenesis work clearly show that ifenprodil and EVT-101 adopt different binding modes. We next speculated as to whether the binding mode of EVT-101 was anomalous or indeed representative of a novel binding motif. Numerous literature compounds do not contain the phenylethanolamine substructure found in ifenprodil but are nevertheless potent and selective GluN2B antagonists (Borza and Domany, 2006; Layton et al., 2006; Mony et al., 2009a). To explore the possibility that GluN2B-selective antagonists can be broken down into subclasses based on binding motif, computational docking methods using our crystallography results were performed to classify 17 literature compounds in addition to the reference compounds ifenprodil, EVT-101 and MK-22. We purposely selected ligands based on their structural diversity and similarity/divergence from the phenylethanolamine pharmacophore found in traditional GluN2B-selective ligands, thus covering both first-generation as well as more recent compounds (Table 2).

**TABLE 2:** Structure of literature ligands used for the in silico docking analysis Activity of each compound is expressed as IC_50_ or *K*_i_ values reported in the literature, obtained using various methodologies (binding assays, electrophysiology).

Compound # (Reference)	Structure	Activity	Compound # (Reference)	Structure	Activity
		*nM*			*nM*
1 (EVT-101) (Kemp and Tasker, 2009)	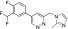	12	11 (Buttelmann et al., 2003)	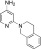	3
2 (MK-22) (Layton et al., 2011)	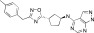	0.9	12 (Alanine et al., 2003)	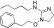	7.5
3 (Ifenprodil) (Carron et al., 1971)	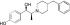	140	13 (Suetake-Koga et al., 2006)	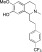	57
4 (Liverton et al., 2004)	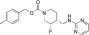	n.d.	14 (Alanine et al., 2002)	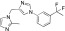	9
5 (King et al., 2013)	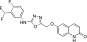	n.d.	15 (Davies et al., 2012)	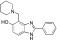	8
6 (King et al., 2013)	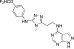	n.d.	16 (Davies et al., 2012)	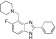	80
7 (King et al., 2013)	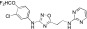	n.d.	17 (Brown et al., 2011)	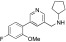	12
8 (Claiborne et al., 2003)	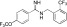	72	18 (Tewes et al., 2015)	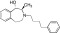	26
9 (McIntyre et al., 2009)	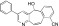	2	19 (Gitto et al., 2012)	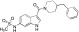	9
10 (CP-101,606) (Chenard et al., 1995)	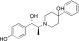	11	20 (Ro25-6981) (Fischer et al., 1997)	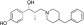	9

**Fig. 4.**
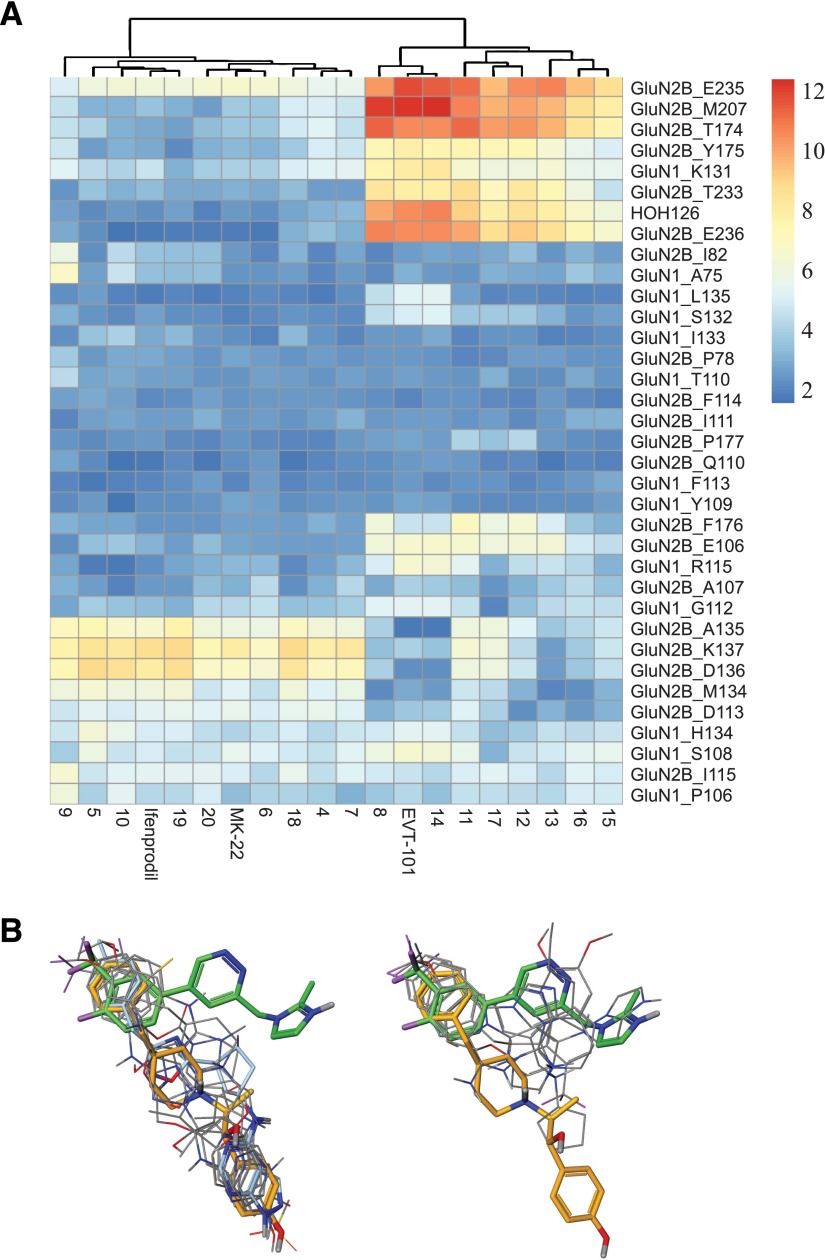
In silico docking analysis of structurally-diverse GluN2B antagonists. (A) Protein-ligand fingerprints ("heatmaps") based on the EVT-101 protein cocrystal structure and displaying computed amino acid-ligand distances. Amino acids are organized according to interaction distance (color code indicates minimal distance to ligands, in Å); numbers on the *x*-axis represent ligands listed in Table 2. Data show two distinct groups of ligands (see arborization on top) indicating at least two main modalities of binding. (B) Pose overlay for the two groups of compounds. Green: EVT-101; orange: ifenprodil. MK-22 depicted as overlapping with ifenprodil on the left in cyan.

Docking of the 20 literature compounds was performed using the structural data obtained either from the EVT-101 (compound **1** in Table 2) or ifenprodil (compound **3** in Table 2) complex. Noticeably, in silico docking experiments reveal two primary clusters of compounds represented by EVT-101 and ifenprodil, irrespective of which structural template was used (Fig. 4 and Supplemental Fig. 3). Compound **14**, for example, which is structurally similar to EVT-101 (**1**), was also found to be a member of the EVT-101 binding mode cluster. This outcome is also true for analogs structurally similar to ifenprodil, such as **10**, **20**, and **4**. The structurally more distant compound MK-22 (**2**) also clustered within the "ifenprodil group", in good agreement with our crystallographic data. Interestingly, compounds such as **8**, **9**, and **11** bear little structural resemblance to the reference ligands, but shared common binding modes with either EVT-101 (compounds **8** and **11**) or ifenprodil (compound **9**). Thus, each group identifies multiple hits, encompassing structurally diverse ligands. In line with our functional results, compounds in the "EVT-101 group" fail to interact with amino acids lining the "bottom" of the NTD dimer cavity (e.g., GluN2B-E235, GluN2B-E236; Fig. 4A and see Supplemental Fig. 2). In contrast, they tended to reproduce the main interactions with the GluN2B NTD *β*4-*β*5 loop (including GluN2B-A135) inferred from the electrophysiology recordings. Also consistent with our structural and functional data, ligands from both groups invariably contacted GluN2B-F114 and neighboring residues, which form a major hydrophobic anchor at the "top" of the NTD dimer interface. Finally, we noted that although compounds in the "ifenprodil group" adopted very similar binding poses, more spreading was observed in the ‘EVT-101 group’ (Fig. 4B and Supplemental Fig. 3B). Compound **14** was in fact the only compound that precisely overlapped with EVT-101, whereas others showed a broader spatial distribution with binding poses intermediate between that of EVT-101 and ifenprodil. It therefore appears that the large cavity at the GluN1/GluN2B NTD dimer interface provides multiple opportunities for ligands to interact with GluN2B NMDARs.

## Conclusions

Decades of medicinal chemistry efforts have led to the identification of a plethora of small ligand compounds acting as selective antagonists of GluN2B NMDARs, some showing therapeutic potential in a variety of neurologic disorders. However, being diverse in their chemical structures, it remained unclear whether GluN2B-antagonists all share a similar binding mode. In the present work, using a combination of high-resolution X-ray crystallography, electrophysiology, and in silico modeling, we map a novel binding site for GluN2B-selective antagonists. This site only partially overlaps with that previously described for ifenprodil and other phenylethanolamine-based GluN2B-selective antagonists, resulting in strikingly different ligand orientation and receptor interactions. We also show that this novel binding modality allows discriminating multiple classes of GluN2B-selective antagonists that differ in their chemical scaffold and binding pose. Thus, GluN2B-antagonists do not form a single homogenous family of drug compounds but segregate into various pharmacophores that target discrete receptor binding pockets. Exploiting this structural diversity should help elucidate GluN2B-selective antagonist structure-activity relationships and facilitate the design of novel NMDAR modulators. It should also open interesting prospects for future drug development to address unmet clinical needs.

## Supplementary Material

Data Supplement

## Abbreviations

NMDAR: *N*-methyl-d-aspartate receptor
NTD: N-terminal domain
