# Supplementary material for: A Novel Binding Mode Reveals Two Distinct Classes of NMDA Receptor GluN2B-selective Antagonists
Source: Mol Pharmacol. 2016 May;89(5):541–51. doi: 10.1124/mol.115.103036 (PMC4859819; doi:10.1124/mol.115.103036)
Supplement: Data Supplement [file supp_89_5_541__index.html]

A novel binding mode reveals two distinct classes of NMDA receptor GluN2B-selective antagonists — A Novel Binding Mode Reveals Two Distinct Classes of NMDA Receptor GluN2B-selective Antagonists — A Novel Binding Pocket for GluN2B Antagonists — Data Supplement 

# A Novel Binding Mode Reveals Two Distinct Classes of NMDA Receptor GluN2B-selective Antagonists

## Data Supplement

**Files in this Data Supplement:**

- Supplemental Data -

  Supplementary Figure 1 - X-ray crystal structure of the GluN1/GluN2B NTD dimer in complex with MK-22

  Supplementary Figure 2 - Two subcavities at the GluN1/GluN2B NTD heterodimer interface

  Supplementary Figure 3 - In silico docking analysis based on the ifenprodil protein cocrystal structure

  Supplementary Table 1 - X-ray crystallography data collection and refinement statistics
