# Supplementary material for: A Novel Binding Mode Reveals Two Distinct Classes of NMDA Receptor GluN2B-selective Antagonists
Source: Mol Pharmacol. 2016 May;89(5):541–51. doi: 10.1124/mol.115.103036 (PMC4859819; doi:10.1124/mol.115.103036)
Supplement: Data Supplement [file supp_mol.115.103036_Supplemental_103036.pdf]

David Stroebel*, Derek L. Buhl*, John D. Knafels, Pranab K. Chanda, Michael Green, Simone Sciabola, Laetitia Mony, Pierre Paoletti§, and Jayvardhan Pandit§; “A novel binding mode reveals two distinct classes of NMDA receptor GluN2B-selective antagonists”, in *Molecular Pharmacology*

Supplementary Figure 1: X-ray crystal structure of the GluN1/GluN2B NTD dimer in complex with MK-22

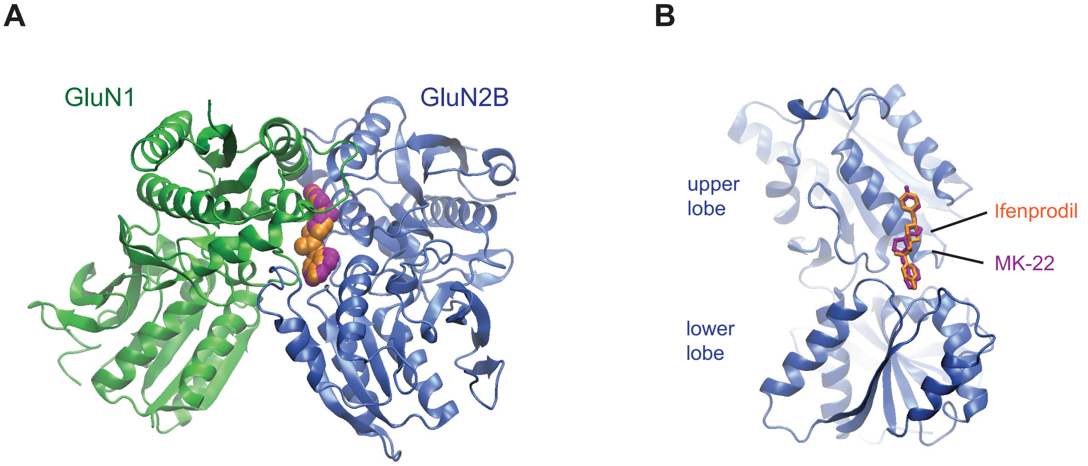

A. Structures of the GluN1/GluN2B NTD heterodimer in complex with MK-22. For comparison purposes, the ifenprodil molecule as seen in the GluN1/GluN2B NTD-ifenprodil complex is superimposed. The two ligands shown in sphere representation (ifenprodil in orange, MK-22 in purple) sit at the heterodimer interface. **B.** Side view (rotated 90°) with the GluN1 NTD removed and the ligands shown in stick representation.

Supplementary Figure 2: Two subcavities at the GluN1/GluN2B NTD heterodimer interface

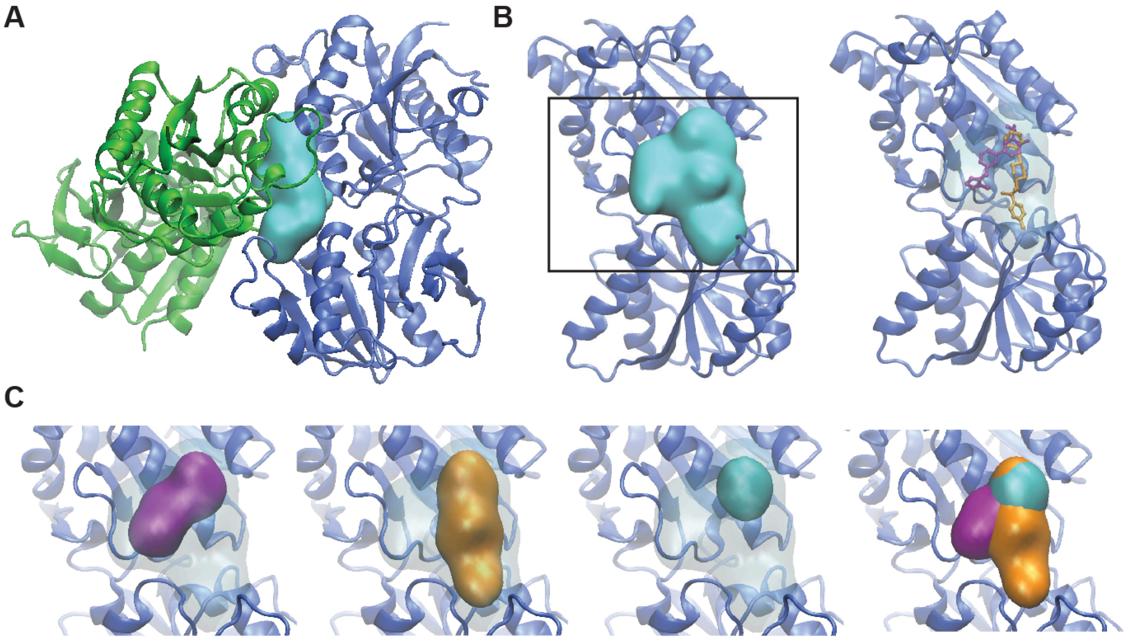

A. Illustration of the cavity at the dimer interface between GluN1 and GluN2B NTDs. **B.** Side view (rotated 90°) with the GluN1 subunit removed. Left: Cavity contour. Right: Ligand poses (EVT-101 in purple, ifenprodil in orange) within the cavity (rendered transparent). **C-F.** The two subcavities. The estimated volume occupied by EVT-101 and ifenprodil are 150 Å^3 (panel C) and 240 Å^3 (panel D), respectively, while the estimated volume of the entire dimer cavity is 400 Å^3 (panel F). The part of the cavity common to both ligands occupies an estimated volume of 50 Å^3 (panel E).

Supplementary Figure 3: *In silico* docking analysis based on the ifenprodil protein co-crystal structure

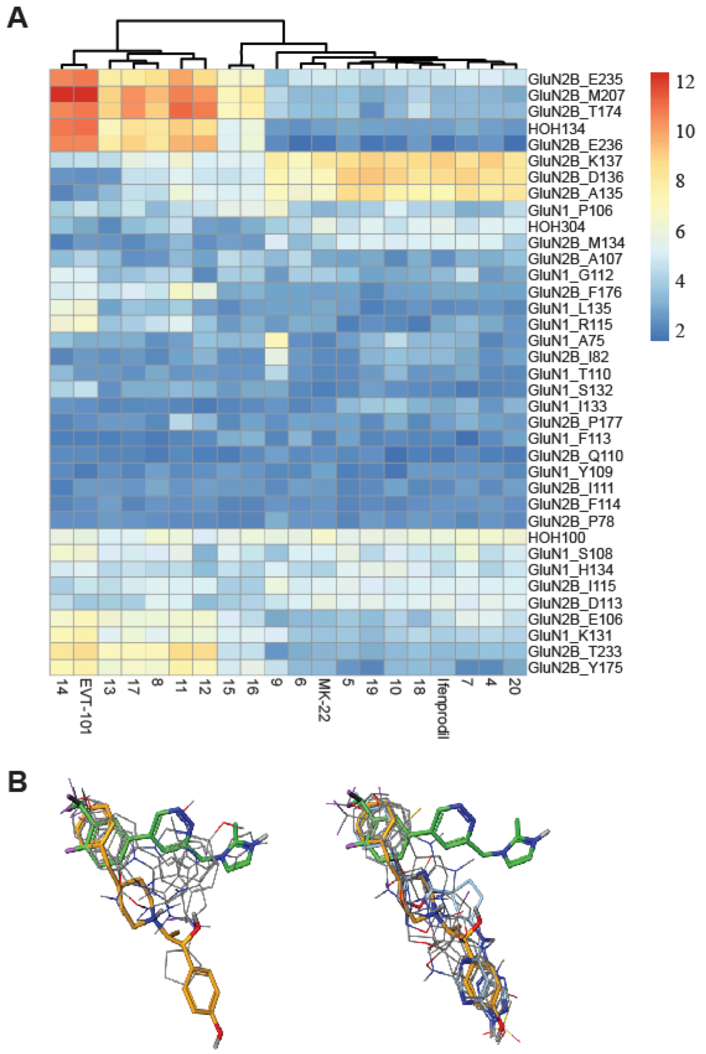

Analysis similar to the *in silico* docking depicted in Figure 4. **A.** Protein-ligand fingerprints (‘heatmaps’) based on the ifenprodil protein co-crystal structure. Amino acids are organized according to interaction distance (color code indicates minimal distance to ligands, in Å); numbers on the x-axis represent ligands listed in Table 2. Data indicate at least two main modalities of binding. **B.** Pose overlay for the two groups of compounds. Green: EVT-101; Orange: ifenprodil. MK-22 depicted as overlapping with ifenprodil on the left in cyan.

Supplementary Table 1: X-ray crystallography data collection and refinement statistics

	EVT-101	MK-22	Ifenprodil
**Data collection**			
Space group	C21	C21	C21
Cell dimensions			
a, *b*, *c* (Å)	268.4, 60.60, 144.5	268.88, 59.94, 144.45	268.46, 60.1, 145.0
α, β, γ (°)	90, 116.27, 90	90, 116.51, 90	90, 116.22, 90
Resolution (Å)	129.57-2.76 (3.9-2.76)*	120.31-2.98(4.21-2.98)	120.42-2.77(3.91-2.77)
R_{sym} or R_{merge}	0.055 (0.140)	0.054 (0.113)	0.056 (0.146)
I / σI	14.1 (7.6)	14.3 (7.5)	17.0 (8.3)
Completeness (%)	98.3 (99.2)	96.2 (95.2)	99.0 (99.7)
Redundancy	3.3 (3.4)	2.6 (2.3)	3.3 (3.4)
**Refinement**			
Resolution (Å)	40.41 – 2.76	30.04 – 2.98	27.92 – 2.77
No. reflections	53264	40941	52883
R_{work} / R_{free}	0.1702 / 0.2137	0.1614 / 0.2140	0.1744 / 0.2102
No. atoms			
Protein	11050	11019	11019
Ligand/ion	486	472	534
Water	290	265	339
B-factors			
Protein	77.3	72.9	75.03
R.m.s. deviations			
Bond lengths (Å)	0.010	0.010	0.010
Bond angles (°)	1.18	1.18	1.19
Ramachandran Plot			
Favored region	1349 (96.2%)	1335 (95.5%)	1342 (95.9%)
Allowed region	44 (3.1%)	59 (4.2%)	46 (3.3%)
Outlier region	9 (0.6%)	4 (0.3%)	11 (0.8%)

*Values in parentheses are for highest-resolution shell.
